# Alternative Medicine and Nutritional Therapy in Adult Inflammatory Bowel Disease in India: A Move Towards Holistic Practice

**DOI:** 10.7759/cureus.116152

**Published:** 2026-09-12

**Authors:** Prerana Ghosh, Karan Varshney

**Affiliations:** 1 Department of Medicine, Peninsula Health, Frankston, AUS

**Keywords:** complementary alternative medicine, crohn's disease, food and nutrition, inflammatory bowel disease, ulcerative colitis (uc)

## Abstract

Inflammatory bowel disease (IBD), including ulcerative colitis (UC) and Crohn’s disease (CD), is a chronic gastrointestinal disorder with increasing incidence in India (also referred to as Bharat), posing a significant public health challenge. Low-cost alternatives to prohibitively expensive long-term pharmacological treatments remain underutilised and unoptimised in treatment guidelines. Such alternatives include complementary and alternative medicine (CAM) and nutritional therapies. For example, herbal medicines, such as curcumin, may be a safe and feasible adjunct therapy and should be considered in patients with UC. CAM practices are prevalent and accepted by patients in India for a wide range of reasons. This narrative review analyses various types of CAM used in IBD patients across India and evaluates data on their efficacy and safety. Additionally, nutritional therapies, like exclusive enteral nutrition, may show promise in managing CD but are not standard practice. We highlight useful recommendations, such as offering different formulations, involving dietitians and trained nurses in a multidisciplinary team to provide patient support, and researching dietary interventions specifically relevant to India. This review further emphasises that CAM and dietary interventions are well regarded and are sought out by patients. Thus, these options should be better understood by practitioners and integrated into practice. By incorporating such practices, India can develop a low-cost, holistic IBD care model that considers the patients’ socioeconomic and cultural contexts.

## Introduction and background

Inflammatory bowel disease (IBD), comprising ulcerative colitis (UC) and Crohn’s disease (CD), is a chronic inflammatory disorder affecting the gastrointestinal tract. Its development is attributed to an interplay of immunological, environmental, and genetic susceptibility [1]. Once thought to be a disease of the West, IBD incidence is now increasing in Asia at alarming rates, partly due to rapid industrialisation and increasing westernised lifestyles and diet [2]. Previously considered low risk, it is now a growing concern in India (also referred to as Bharat), with an incidence of 9.31/100,000, the highest rate in Asia. Being highly populous, India also has the second-largest IBD population globally after the United States [3]. Due to its chronicity and low mortality, disease prevalence is expected to increase in India as the country grapples with an ageing population and rising global incidence. IBD impairs quality of life (QoL) and functional capacity, leading to high costs associated with morbidity and treatment [4,5]. Therefore, over the coming decades, it is forecasted to be a major public health and economic burden for India. While access to diagnostic services is improving, especially in rural areas, there are still therapeutic challenges with large disparities in income and affordability.

IBD treatment is expensive, encompassing healthcare costs, productivity loss, caregiver costs, and patient costs [6]. Estimated annual patient costs in India during disease remission range from USD $527 to $940 [7]. This can increase up to USD $807 to $1512 during active disease, with medications being the primary contributor [7]. These costs in India are still substantially lower than costs in Europe or North America, where hospitalisation increases expenses [6,7]. Despite this, in India, there is a proportion of the population with uncertainty pertaining to the availability of care due to being uninsured. The proportion of uninsured patients is variable, and even in those who are insured, only 18% of the total cost is covered by insurance [7,8]. This presents a challenging and unsustainable model of IBD care where lifelong monitoring and treatment are required for frequent disease relapses [9]. Many patients, especially from impoverished rural backgrounds, will be unable to afford multiple consultation fees, let alone medicinal treatment [8].

As active disease is more expensive, alternative strategies aimed at minimising risk of flares and maintaining remission should be explored. Given resource limitations and inadequate government healthcare insurance policies, management guidelines from high-income countries are not completely transferable or sustainable in the context of India. Hence, it is pertinent to explore the efficacy of low-cost alternatives, including complementary and alternative medicine (CAM) and nutritional therapy in the treatment of IBD, which are currently either unoptimized or underutilised [3,10]. CAM refers to forms of medicine that are outside the realm of conventional biomedicine and are wide-ranging, encompassing practices such as Ayurvedic medicine and Chinese traditional medicine (Chinese TM). This narrative review aims to put forth evidence-based recommendations and tailored approaches that can improve patient care in India, while advocating for a more holistic patient experience. More precisely, it aims to focus on the underutilised role of CAM in IBD care in the country, and the challenges that limit the extent of its usage. In addition, we highlight the high value of focusing on dietary interventions to improve IBD care in the country, particularly with regard to exclusive enteral nutrition (EEN).

## Review

Methodology

For this narrative review, we aimed to focus on assessing and synthesizing a number of aspects of the available literature. First of all, we focused on current, conventional pharmacological management of IBD in India. Next, we evaluated CAM in the context of IBD both globally and specifically within India. This included a brief portion on perceptions regarding CAM, but we primarily focused on the forms of CAM used in IBD management in India. We evaluated the strongest forms of evidence available from trials, but where this data was not available, we evaluated other sources of evidence, such as cohort studies, as well as animal studies and case series/reports. Secondary sources were also utilised, where relevant. After synthesizing this evidence, we evaluated the landscape of the literature with regard to current challenges when it comes to the integration of CAM in IBD care in India; moreover, we synthesized the evidence to provide recommendations for the next steps forward.

The next part of the narrative review focused on the utility of nutrition-based management in IBD in India, with a particular focus on exclusive enteral nutrition (though the analysis is not limited to this). Based on the synthesis of this literature, and further literature regarding the application of EEN in India for IBD, we discuss challenges and provide further recommendations regarding EEN and CAM implementation in India more generally.

In this narrative review, we evaluated published studies by searching numerous databases with the intention to provide the most up-to-date literature. This included searches in Embase, PubMed/Medline, and Google Scholar. When required, studies were also searched and assessed via ResearchGate, as well as via grey literature. Reference lists of prior reviews were also evaluated to find additional, relevant articles. Search terms were intended to be broad to include as many relevant articles as possible. Search terms typically pertained to CAM, specifically Ayurveda and nutrition (such as EEN), in relation to IBD and India.

Current pharmacological landscape for IBD treatment in India

Aminosalicylates and corticosteroids have been the mainstay of treatment in IBD management for both induction and maintenance of disease remission [11,12]. Aminosalicylates, the most commonly prescribed drugs in IBD, are cheaper than biologic therapy [13,14]. Thiopurines are also low-cost immunomodulatory drugs that are effective in maintaining steroid-free remission in IBD and preventing postoperative relapse in CD [11]. Despite being cheap and widely prescribed, non-adherence to the IBD medication regimen is higher in India than in Western cohorts [9]. Reasons for non-adherence include lack of belief and adequate medication information, drug side effects and efficacy, high-cost perceptions, and reduced QoL [9]. Non-adherence increases risk of relapse, increases healthcare costs, and worsens QoL [9,15]. Thiopurine use is also associated with the development of thiopurine-induced leukopenia that further exacerbates treatment costs and loss of productivity [11].

Biologic therapy, consisting of anti-TNF inhibitors and anti-integrin inhibitors, has revolutionised the management of IBD [11]. It is estimated that 10% of UC and 10-15% of CD patients require biological therapy in India [16]. In reality, very few patients can afford maintenance biologics, as the estimated annual patient cost is $20,000-22,000 [14]. Due to inadequate health insurance coverage and expensive private health insurance, the proportion of patients in India receiving biologics is just 1.5-1.8% [2,8,13]. In contrast, in other Asian countries like Japan, where the government covers biologic expenses, around 40% of patients receive biologic therapy [14].

There are additional challenges of long-term pharmacological treatment. Physician hesitancy to prescribe immunomodulators and biologics stems from concerns of latent tuberculosis reactivation, especially in India where tuberculosis is highly prevalent and endemic [17]. Newer biologics do not increase reactivation risk, but these are not currently available in the Indian market [11,18]. Patients also have a general distrust in modern conventional medicine, with acceptance failure due to fear of adverse effects and the chronic requirement of medications [19]. Additionally, unique cultural taboos and stigma favour the use of CAM as primary treatment modalities in many places [20]. These practices and perceptions are further bolstered by the CAM industry’s advertising of adverse effects of conventional medicines [20].

CAM in IBD

Worldwide, people seek holistic healthcare practices, like CAM, for prophylactic and therapeutic reasons. CAM is a diverse group of medical practices that are not integrated into conventional medicine, encompassing herbology, dietary supplementation, and mind-body techniques like acupuncture [11,21]. CAM use is prevalent among IBD patients globally, with herbal remedy use being the most commonly reported [22,23]. In India, CAM practices, such as homeopathic and Ayurvedic therapies, are widely used, especially among chronic disease patients [11,24-27]. A high proportion of rural populations in India rely on CAM as their primary healthcare [21].

Patients’ faith in CAM is due to its long and rich history in India and the belief that traditional medicine has fewer side effects than conventional medicine, which may also influence its self-administration [24]. IBD patients prefer CAM due to perceptions that CAM is more effective than conventional medicine, dissatisfaction with conventional therapies, and having a feeling of control over their disease [22,26,28-30]. Socioeconomic factors can also influence uptake of CAM. CAM usage was significantly associated with not being covered by any health insurance scheme [31]. Hence, patients use CAM as it is cheaper and easily accessible [30].

An extensively studied CAM option in IBD is curcumin, a phytochemical active agent of turmeric, traditionally used in Chinese TM and Ayurveda [23]. Curcumin has been shown to have anti-inflammatory and anti-oxidative effects [11]. Several double-blinded, randomised controlled trials (RCTs) have demonstrated curcumin to be a feasible and safe adjunct therapy in achieving disease remission, improving disease activity, and maintaining remission in mild-to-moderate active UC [32-36], although these studies had small sample sizes, short study durations, lack of power calculations, and heterogeneity in curcumin dosages and formulations, and were limited to just mild-to-moderate disease [32,34-36]. A subsequent meta-analysis found significantly higher rates of disease remission with curcumin [23]. Recently, a larger RCT combined curcumin with another herbal compound and corroborated the findings from earlier RCTs [37]. Currently, curcumin shows positive results in UC and can be an affordable option, or an add-on to treatment, in settings where it may help to reduce the healthcare burden [11,22].

Evidence regarding curcumin efficacy in CD is limited and conflicting [38,39]. Nonetheless, it is important for physicians to be aware of this, so that they may advise their patients accordingly. The evidence pool pertaining to CAM use in CD may improve in the future with better knowledge about plant-derived natural products, widespread utilisation of Ayurvedic practices by allopathic physicians, and development of appropriate research methods to test their efficacy.

There are a number of different examples of the application of CAM, particularly in the context of Ayurveda, to manage IBD. One RCT focused on the herbal treatment known as *Pathadya Churna* in the management of UC. The study found that, compared to the standard treatment, those receiving *Pathadya Churna* had greater improvements in digestive outcomes, appetite, general well-being, and disease activity [40]. Another study focused on combining Ayurvedic medications with standard biomedical treatment, where the Ayurvedic treatment of *Udumbara kvatha basti* (which contains an array of different herbs) was administered alongside conventional steroids in UC patients. It was found that there was more than a 75% reduction in inflammation in those treated with the combination approach as opposed to those strictly on steroids [41].

Another form of CAM treatment (Ayurvedic) known as *Triphala *has also shown some promise in reducing colonic inflammation in IBD occurring in rats [42]. In another study in mice with IBD, a polyherbal suspension containing Ayurvedic formulations of *Burma dhaniya* (*Eryngium foetidum*), *Sapota *(*Manilkara zapota*), and Curry leaves (*Murraya koenigii*) was assessed for its effect against IBD, and reductions in inflammation and oxidative damage were seen in the mice [43]. There are also a number of small-scale studies in the form of case reports that describe significant improvements in outcomes, and even remission, for IBD patients using Ayurvedic medications [44-46]. While some of the evidence base is clearly weaker than others based on small sample sizes, heterogeneous approaches, and being limited to animal models, the available literature does nonetheless highlight potential in a number of areas for CAM to have a useful role in IBD management in India. An overview of the evidence supporting these Ayurvedic approaches is shown in Table 1.

**Table 1:** Overview of evidence for CAM (specifically, Ayurvedic approaches) in IBD management CAM: complementary and alternative medicine; IBD: inflammatory bowel disease; UC: ulcerative colitis; CD: Crohn’s disease

Type of treatment	Impact	Source of evidence
Curcumin	Feasible, safe adjunct in UC therapy by helping in achieving disease remission, improving disease activity, and maintaining remission in mild-to-moderate active UC. Additional evidence highlighting its usage can improve remission rates; limited and conflicting evidence in CD [38,39]	Randomised controlled trials [32-37]
Pathadya Churna	Compared to the standard treatment, those receiving *Pathadya Churna* had greater improvements in digestive outcomes, appetite, general well-being, and disease activity	Randomised controlled trial [40]
Udumbara kvatha basti	More than a 75% reduction in inflammation for those treated with the combination approach as opposed to steroids alone	Randomised controlled trial [41]
Triphala	Reduced colonic inflammation	Animal study [42]
A polyherbal suspension containing Ayurvedic formulations of *Burma dhaniya*, *Sapota*, and Curry leaves	Reductions in inflammation and oxidative damage	Animal study [43]
A personalized regimen of *Deepana-Pachana* included *Kutajghan Vati*, *Panchamrit Parpati*, *Bilwadi Avaleha*, and *Manasamitra Vati*	Complete symptomatic remission in 3 months, with complete mucosal healing	Case report [44]
*Drakshovin*, *Manoll*, and *Narasimha Rasayana*	Remission of disease	Case report [45]
A personalised regimen with 15 different Ayurvedic therapeutic interventions	Remission after 1 year, with recurrence of symptoms at a later point	Case report [46]

Challenges and recommendations for CAM use

A major challenge is that patients utilising CAM do not seek their conventional medicine doctor’s or CAM practitioner’s approval before using CAM. To date, there is limited evidence regarding the extent of integration, or lack thereof, for CAM specifically in the context of IBD care. However, there does exist relevant evidence in other contexts. For example, a pan-India study (in the context of COVID-19) reported that around 47% of patients self-administer CAM and only 18.6% consult CAM practitioners [24]. Moreover, patients consult non-trusted sources like family and friends [30]. This raises concerns regarding patient safety, drug-drug interactions, and increased risk of adverse side effects in patients with concomitant comorbidities. Using certain types of CAM products increases the risk of flares in UC, which would decrease QoL and further increase treatment costs [15]. Communication regarding CAM use between physicians and patients is also poor, and often treating physicians do not enquire about CAM usage in their history-taking [15,26]. Therefore, first, IBD physicians should use the opportunity of diagnostic consults to explore CAM practices in their patients and optimise their use. Second, they should counsel patients on appropriate options and their safety profile through evidence-based practice on a case-by-case basis.

Many physicians lack the training, knowledge, and access to high-quality clinical data required to guide their patients on CAM use despite the Government of India launching the Ministry of AYUSH (Ayurveda, Yoga, Unani, Siddha, and Homeopathy) to promote awareness of medical pluralism [21,28,30,47]. Types of CAM and reasons for using them are heterogeneous among populations. Although CAM usage in India is prevalent, evidence on what proportion of IBD patients in India use CAM, the types of CAM used, and their underlying reasons is currently lacking. Therefore, profiling the use of CAM in IBD patients in India should be done to better inform IBD physicians on their patients’ behaviours. There is also wide variation in quality and inconsistencies in the evidence regarding the efficacy and safety of different CAM modalities in IBD, which hinders their inclusion into clinical practice [11,23]. This warrants further research, especially for those options that are frequently being used by IBD patients in India. Many patients are interested in participating in studies integrating Ayurveda with modern medicine [26]. Hence, larger clinical trials exploring the efficacy of various types of CAM in IBD should be conducted to improve the quality of the existing literature, with the aim of setting up standardised guidelines. Such evidence would be crucial for physicians to guide their patients through the ‘CAM jungle’ and protect patients from ineffective and unsafe practices. Additionally, the government and medical education boards should incorporate CAM in the mainstream medical curriculum to enhance knowledge, advance professional qualifications, and standardise formal training [22].

India has a wealth of traditional and alternative medicine systems available, but their integration into conventional medicine has been limited. Studies have shown that many doctors believe in CAM and feel that integrating them into conventional modern medicine would be effective [25]. While many patients consult Ayurvedic physicians for advice, they are either hesitant or do not disclose their Ayurvedic practices to treating physicians over concerns that they may be treated differently or asked to stop [26]. This highlights the disconnect in attitudes, practice, and integration. Although many patients believe that it would be beneficial if their tertiary healthcare institute had Ayurveda services on offer [26], to our knowledge, dedicated AYUSH departments do not exist at every tertiary or primary healthcare service in India. In contrast, 90% of all hospitals in China have set up TM departments [48]. Therefore, it is imperative for IBD physicians to collaborate, refer, and share holistic patient care with registered AYUSH practitioners [49]. Hospitals across India should attempt to set up Ayurvedic departments and scale up existing integrative approaches. It is also important, however, to recognise the limits of these approaches, and their development should occur alongside the ongoing scaling up of conventional, biomedical infrastructure and staffing.

Merits of nutritional therapy in IBD in India

An additional element of care linked to the previous discussion regarding CAM revolves around the utility of dietary interventions in IBD management throughout India. Diet contributes to IBD pathogenesis by driving inflammation and gut microbiome dysbiosis [50]. IBD’s rise in developing countries like India is partly attributed to westernised diets (low fruit and vegetable intake, and a high intake of fat, animal protein, and refined sugars) [50]. Thus, dietary interventions are a safe physiological strategy to manage IBD-associated inflammation while improving nutritional status in patients. Patients in India tend to associate diet with chronic conditions. A survey found that nearly 85% of respondents associated faulty diet and lifestyle with the development of diseases [26]. Hence, using dietary interventions in IBD management has a logical basis.

While various diets have been trialled in IBD patients, exclusive enteral nutrition is the most researched. EEN is a low-cost, low-risk and minimally invasive therapy that provides the daily nutritional requirement completely through a liquid formula taken orally or by a feeding tube for a six-to-eight-week period [11,51]. With up to 80% remission rates, it is internationally recommended as a first-line induction and remission therapy in paediatric CD [11,52]. Its proposed mechanism is through direct and indirect anti-inflammatory effects, restoring the diversity and balance of the gut microbiome and correcting nutritional deficiencies [53]. Emerging evidence highlights its role in adult IBD management as well [53].

EEN is effective at improving clinical outcomes while reducing pharmacological side effects. While an earlier Cochrane review found EEN to be inferior to corticosteroids for inducing remission, the evidence was of low quality, heterogeneous, and dependent on patient adherence [54]. With high rates of patient adherence, EEN had no significant difference in remission rates compared to corticosteroids but had a better safety profile [54]. A later systematic review, meta-analysis, and meta-regression confirmed that EEN improves disease activity and induces disease remission [55].

Beyond remission, EEN also has better long-term outcomes. Compared to corticosteroids, EEN has higher rates of endoscopic mucosal healing that reduces the risk of flares and surgery requirements [53,56]. It also reduces postoperative adverse effects in adult CD. Preoperative EEN use significantly reduced postoperative complications, compared to corticosteroids [53,57]. EEN may also have a role in CD maintenance therapy. Partial EN (PEN) consists of supplying half of the patient’s caloric intake as EEN, and the remainder from a whole-food diet. Compared to an unrestricted diet, PEN is associated with high rates of remission among adult CD patients [53]. Another systematic review found an association between PEN and induction and maintenance of remission in CD [58].

In India, a small number of studies have been conducted on the usage of EEN in IBD management. One study with only 23 patients demonstrated that EEN was effective, and well-tolerated, in inducing remission in adult CD that was refractory to anti-TNF [59]. Similar findings were demonstrated for an analysis including 31 patients receiving EEN for two to six weeks [60]. Of pertinence, one RCT conducted in a population in India has shown that EEN can improve responses to intravenous corticosteroids in acute severe UC patients in comparison to steroids only [61]. An additional small-scale study conducted in this context has demonstrated that EEN with corticosteroid usage can improve the gut microbiome of patients with acute severe UC [62].

Challenges and recommendations for implementing EEN

Despite its potential for high utility, it appears that EEN for IBD is under-prescribed in India. For example, a prospective multicentre study on paediatric IBD in India, which included 325 children, demonstrated that EEN is underutilised across the country; though this study specifically focused on a paediatric population, it is nonetheless relevant to highlight the extent to which EEN is underused [63]. A study on adult IBD demonstrated that undernutrition is prevalent in as many as 27% of IBD patients [64]. Therefore, it is instructive to learn from other contexts where its usage is more widespread. In Japan, EEN is recommended for induction and maintenance of CD remission [65-67]. A significant barrier underpinning its use is poor patient acceptance and adherence, leading to high non-compliance rates which limit its effectiveness [51,53,60]. Possible reasons include its unpalatable taste, lack of variety, missing out on social aspects of food, lack of motivation, lack of acceptance of a nasogastric tube (NGT), inadequate patient support, financial costs, and length of treatment [67]. It is also impractical to continue patients on an exclusively liquid diet for six or more weeks, as compliance rates reduce with longer durations of treatment [53]. Thus, it is challenging to use EEN as maintenance therapy.

Despite these challenges, several solutions have been put forth. Unpalatability may be mitigated with different formulations. Currently, there are three available formulations with no significant difference in efficacy [54]. The polymeric formula is, however, more palatable and may, therefore, be better tolerated [68,69]. This formula is also cheaper compared to others [60]. Another way to overcome adherence issues is by providing support and education, and reducing therapy duration. High rates of adherence were achieved when patients received in-patient EEN with support from nurses and nutritionists and had shorter durations of therapy [53,70]. These services could be offered to patients, especially in the initial or final weeks of therapy, as adjustment difficulties usually occur during these periods [68]. The government could be pivotal in setting up IBD hubs offering such services in CD-prevalent areas. Additionally, educating patients on the benefits and mechanism of EEN, clear instructions on how to commence the diet, strategies on managing common challenges along the way, and setting expectations about adjustment periods and experiences also improved compliance [68,70,71]. This is especially pertinent considering that, in one study, with appropriate dietary advice and optimisation of therapy, nutrition improved in 53.7% of participants [64]. Therefore, with appropriate education programs, trained staff to provide nutritional guidance, and necessary in-patient infrastructure, EEN compliance rates can be improved.

Beliefs about what is beneficial in reducing symptoms of IBD can influence dietary decisions, including self-imposed dietary restrictions. An observational study found that while a large proportion of surveyed patients had modified their diet since their IBD diagnosis, including self-imposed dietary restrictions, only <50% of them actually received dietary counselling [72]. The need for involving dietitians in IBD care and increasing physician awareness and training to manage the same is a key consideration. Patients often exclude multiple food items from their diet when improperly counselled and endure malnutrition, which is not only further exacerbated by their disease course but also influences disease prognosis [53,73]. Individual needs of a patient should be considered when counselling them on the challenges of diet restriction and risks associated with nutrient deficiencies, highlighting the need for collaboration between physicians and dietitians [74]. Therefore, a multidisciplinary approach is recommended when starting patients on EEN, also extending it to other IBD patients to improve patient satisfaction and decrease healthcare costs [75,76]. Ultimately, development of a patient-specific plan involving dietitians is essential.‌

The shortcomings of a one-size-fits-all approach should also be acknowledged. Not all patients can tolerate drastic changes to their diet and lifestyle. Social impacts and the monotonous nature of EEN also pose a challenge to acceptance [68]. Alternative diets such as the CD-TREAT diet (this recreates EEN but is personalised to patients), CD exclusion diet (CDED), and PEN may have a role in IBD management [65,76]. Clinical studies have shown similar efficacy of CDED and CD-TREAT to EEN but better tolerability and compliance rates than EEN [76,77]. Studies on their efficacy in the adult IBD population of India remain scarce, and hence, these options should be explored in larger RCTs to strengthen their recommendations in the clinical guidelines. Additionally, studies on the influence of societal factors, experiences, and patients’ perceptions towards EEN uptake in India remain limited. Sociocultural factors governing dietary choices should be considered by research and industry stakeholders when developing further formulations, flavours, and textures to address palatability and lack of variety issues. Additionally, it is important for physicians to acknowledge the social impact of EEN and counsel patients on appropriate coping strategies, including enhancing patients’ support networks to improve adherence [68]. In addition to these challenges, it is also worthwhile to denote that much more research is required on the effectiveness of CAM treatments; while some may require conventional studies in the form of RCTs to most effectively highlight the extent of the evidence, in other contexts it may be especially useful to build on the existing research in other domains. For example, researchers have focused on this in the context of diabetes outcomes and the link between the gut microbiome and mental health [78,79]. Replicating and building on such research in relation to CAM for IBD may allow for greater insights into the holistic benefits of these treatments.

## Conclusions

The increasing incidence of IBD in India poses economic and public health concerns, necessitating low-cost alternatives in light of challenges with affordability and access to pharmacological treatment. Both CAM (through approaches such as those from Ayurvedic medicine) and EEN are affordable, low-risk, and safe alternatives showing promising results in IBD management, and may offer utility alongside (or, in some cases, in place of) pharmacological treatments. Despite their potential, they are not being optimised or utilised effectively due to numerous barriers limiting their implementation in clinical practice. Changes can occur across epidemiological research, clinical trials, health services, and government sectors. Integrating these options into practice may help India potentially lower the cost of treatment for both patients and the healthcare system; however, more research is needed in this area. In conclusion, domains of public health and research may warrant using holistic models of care in IBD management to more comprehensively support the patient journey.
